# Service Evaluation of the Impact of Capnography on the Safety of Procedural Sedation

**DOI:** 10.3389/fmed.2022.867536

**Published:** 2022-05-06

**Authors:** Gareth Corbett, Peter Pugh, Jurgen Herre, Teik Choon See, David de Monteverde-Robb, Rafael Torrejon Torres, Rhodri Saunders, Catherine Leonard, Amit Prakash

**Affiliations:** ^1^Department of Gastroenterology, Cambridge University Hospital National Health Service Trust, Cambridge, United Kingdom; ^2^Department of Cardiology, Cambridge University Hospital National Health Service Trust, Cambridge, United Kingdom; ^3^Department of Respiratory Medicine, Cambridge University Hospital National Health Service Trust, Cambridge, United Kingdom; ^4^Department of Radiology, Cambridge University Hospital National Health Service Trust, Cambridge, United Kingdom; ^5^Department of Pharmacy, Cambridge University Hospital National Health Service Trust, Cambridge, United Kingdom; ^6^Health Economics, Coreva Scientific, Koenigswinter, Germany; ^7^Health Economics, Medtronic UK Ltd., Watford, United Kingdom; ^8^Department of Anaesthesia, Cambridge University Hospital National Health Service Trust, Cambridge, United Kingdom

**Keywords:** safe sedation practice, monitoring, respiratory compromise, endoscopy, bronchoscopy, patient safety

## Abstract

**Background:**

Capnography has been associated with a reduced incidence of events related to respiratory compromise during procedural sedation.

**Methods:**

A prospective service evaluation was conducted at a large United Kingdom (UK) teaching hospital to assess the impact of capnography on patient safety within four speciality services: bronchoscopy, endoscopy, interventional cardiology, and interventional radiology. Events were defined as provided by the World Society of Intravenous Anaesthesia. One thousand four hundred one patients were enrolled in the evaluation, with 666 patients before and 735 after implementation of capnography. Data was entered as a convenience sample on site in an offline data-collection tool. Results were assessed for the relative reduction in the incidence and resulting adjusted odds ratio for the combined incidence of oxygen desaturation (75–90% for <60s), severe oxygen desaturation (<75% at any time) or prolonged oxygen desaturation (<90% for >60s), bradycardia (>25% from baseline) and tachycardia (>25% from baseline). The adjusted odds ratio was controlled for both procedure and patient characteristics.

**Results:**

After implementation of capnography, a significant reduction (43.2%, *p* ≤ 0.05) in adverse events was observed: 147 adverse events occurred during 666 procedures without capnography compared with 93 adverse events that occurred during 735 procedures with capnography. The adjusted odds ratio for the occurrence of the target adverse events was 0.57 (95% CI: 0.42–0.77). Multivariable linear regression indicated that capnography was a significant predictor (*p* 0.001) of reduced adverse events.

**Conclusion:**

These results suggest improved patient safety following capnography implementation.

## Introduction

Procedural sedation is an established method within the English National Health Service (NHS) to maintain patient comfort during healthcare interventions that do not require general anesthesia. Sedation puts patients at increased risk of impaired ventilation, which may result in respiratory compromise. Although generally considered a rare event, prospective clinical data indicate that respiratory compromise is more common than incidences frequently reported in retrospective analyses (1, 2). In contrast to several other European countries, where an anaesthesiologist is present to guide procedural sedation, procedural sedation in the United Kingdom (UK) is commonly conducted and supervised only by the attending physician. This means that the physician both performs the procedure and ensures that the patient is safely sedated. The UK practice of physician-led sedation was only superficially described in the latest European Society of Anaesthesiology (ESA) guidance (3). Notably, the ESA did not include physicians from the UK in the panel, sparking criticism from UK physicians (4). It is, therefore, questionable whether the ESA provides sufficient guidance relevant to safe sedation in the UK.

The most common procedures utilizing procedural sedation in the NHS are those of gastroenterology. For example, endoscopy, requiring procedural sedation, accounts for more than 2.5 million procedures annually within the UK (5). A common concern during complex and prolonged procedures is oversedation (5). The British Society of Gastroenterology found that 33% of patients undergoing endoscopic retrograde cholangiopancreatography (ERCP) with procedural sedation received more than 5.5 mg of midazolam (6). Of these procedures, 8% required reversal agents to complete the procedure (6). In a survey of hospital commissioners and providers of procedural sedation, hypotension and bradycardia were the two most frequently reported sedation-related adverse events (7).

Reducing sedation-related adverse events was identified as an action point for the quality of care at Cambridge University Hospitals NHS Foundation Trust (CUH) and was a high-level priority of the local multidisciplinary Safe-Sedation Committee at CUH. Given the evidence supporting capnography use (1–3, 6, 8–10), the Safe-Sedation Committee at CUH were keen to introduce capnography monitoring but in a manner that precluded indiscriminate use of this technology. The aim was to understand and focus its use on preventing complications in procedures most likely to be associated with them. As part of a service evaluation (SE), the Safe-Sedation Committee at CUH recommended exploring the addition of capnography monitoring to the required monitoring modalities during procedural sedation. The SE was undertaken in four services: bronchoscopy, endoscopy, interventional radiology (IR), and interventional cardiology (IC).

In undertaking a SE of capnography monitoring, the objective of the Safe-Sedation Committee was to quantify the impact on patient safety and consider the cost-benefit of including capnography as a commissioned element of local procedural sedation guidelines.

## Methods

### Initial Practice and Hospital Perspective

During procedural sedation, the defined minimal monitoring standards at CUH are: pulse oximetry (SpO2), electrocardiogram (ECG), and non-invasive blood pressure (NIBP) (8). Capnography is already in use at CUH, where it is an accepted technology for monitoring during a subset of procedural sedations and mandated by guidelines if general anesthesia is used (5).

### Registration and Reporting Standards

CUH supported the integration of capnography monitoring as an established, non-experimental element of procedural sedation. There was no prospective patient assignment or randomization, and so the addition was classified as a SE and not a clinical study. On assessment by the participating departments, ethics committee approval was deemed not required but the evaluation project is registered with CUH's Clinical Audit department and results reported in line with their requirements.

### Service Evaluation Design

This SE was conducted in the bronchoscopy, endoscopy, IR, and IC departments. The full list of procedures included in this SE are available in Supplementary Tables S1–S5. To evaluate the impact of capnography monitoring on practice at CUH and to allow for potential risk-stratified implementation after the SE, data before and after introduction of capnography were collected and analyzed. Inclusion criteria were all adult patients classified as American Society of Anesthesiologists (ASA) I–IV, scheduled for a healthcare intervention using procedural sedation within one of the participating departments. Patients were selected as a convenience sample, dependent on whether staff were available to log and report on intra-procedural adverse events. The capnography monitor in use was the Medtronic Capnostream technology which was provided free of charge by the manufacturer during this SE. Group training on device setup and identifications of deviations was provided by the sponsor on-site and virtually from the outset of SE.

### Data Collection

Patient parameters known to potentially influence the adverse-event risk were identified from the literature and included in the SE data collection. The parameters recorded were: procedure time, procedure type, sedative chosen, patient ASA classification, clinician ID, use of capnography, use of supplemental oxygen, peri-procedural adverse events and interventions, escalation of care, and patient death. Data collection was performed by nursing staff utilizing paper forms to be later entered in an on-site, offline Microsoft^®^ Excel™-based form by nursing staff. The data was entered in a pseudonymized fashion with no direct patient identifiers recorded. The data entry form was encrypted and secured by password protection.

Adverse events and interventions were available as discrete events for selection and chosen from the World Society of Intravenous Anaesthesia (SIVA) adverse event reporting tool (11). Low, moderate, and high risk classifications used within the scope of this manuscript correspond with the minimal, low, and sentinel risk classifications as introduced by Mason et al. (11). The following adverse events could be selected on the data-collection form: oxygen desaturation (75–90% for <60 s), severe oxygen desaturation (<75% at any time) or prolonged oxygen desaturation (<90% for >60 s), prolonged apnoea (>60 s), airway obstruction, bradycardia (>25% from baseline), tachycardia (>25% from baseline), cardiovascular shock/collapse, cardiac arrest/absent pulse, and “other”. Interventions available were pre-defined as: chest compressions, tracheal intubation, oral/nasal airway, bag valve mask/assisted ventilation, laryngeal mask airway, continuous positive airway pressure (CPAP), use of reversal agents, and “other”.

### Primary Outcomes

The primary endpoint was defined as the total incidence of the following adverse events: oxygen desaturation (75–90%) for <60 s, severe oxygen desaturation (<75% at any time) or prolonged oxygen desaturation (<90% for >60 s), bradycardia (>25% from baseline), and tachycardia (>25% from baseline). One patient could experience multiple adverse events but multiples of the same event in a single patient were only counted as one event. As a hypothetical example, a patient experiencing two oxygen desaturation (75–90%) events for <60 s and a single tachycardia event during the procedure would be counted as having two adverse events. Incidence refers to the number of adverse events per 100 procedures.

### Group Size Calculation for Achieving Significance

This SE aimed to quantify the effect of the addition of capnography to standard procedural sedation practice at CUH. A 20% reduction in the primary endpoint was considered clinically meaningful by the Safe-Sedation Committee. The cumulative baseline incidence (30.48%) for the target adverse events was estimated utilizing the individual event incidences reported in prospective studies analyzed by Saunders et al. (12). To power the SE, the required minimum patient count to fulfill the combination of the effect size and the Type I (10%) and Type II (20%) error criteria was calculated to be 666 patients per group (13). The acceptable Type I error was set to 10%, which corresponds to a 90% chance that rejection of the null hypothesis would be a true positive finding: i.e., that a 20% reduction in adverse events is attributable to capnography monitoring. The Type II error was set to 20%, meaning that there is 80% chance that accepting the null hypothesis would be a true negative finding.

### Data Analysis

The population data were analyzed for differences between groups and the consistency of the recorded data. Groups were compared for consistency using the Mann-Whitney test for categorical data and the Chi-squared test for binomial data. The difference between groups with respect to the primary outcome was assessed using risk ratios. Risk ratios were also calculated for adverse events and interventions. Sub-group analyses were performed by stratifying data by: (1) department, (2) sedative agent, and (3) ASA classification.

### Potential Confounders

As this SE used a convenience sample without randomization, there is potential for the baseline and capnography groups to show differences in the distribution of patient and procedure recorded parameters. We considered the following recorded parameters to be potential confounders in the analysis if they were imbalanced between the two groups: department, sedation level, ASA classification, use of capnography, and use of supplementary oxygen.

A multivariable regression model was created to estimate the impact of recorded parameters on the adverse event occurrence. The model used a binomial error distribution with the recorded parameters as its predictors. Calculations were performed using R version 4.0.0. with the built-in “stats” package, version 4.0.0. Adjusted odds ratios were calculated using the “oddsratio” package, version 2.0.0. Statistical significance was defined as *p* <0.05; decisions regarding clinical significance are made at the reader's discretion.

### Protocol Deviations

During the SE, the IR department discontinued data reporting in March 2018 during data collection for the pre-capnography (baseline) group. The IR department determined that they would continue using capnography monitoring for procedural sedation outside of the SE. As data reporting by IR was discontinued before completion of the SE, the data from the IR department are not analyzed here. Therefore, results represent data from endoscopy, IC, and bronchoscopy.

## Results

Data collection for procedural sedation without capnography took place between December 2017 and October 2018, followed by data for procedural sedation with capnography collected between October 2018 and January 2020. For the baseline group, 666 patients were recorded, while for the post-capnography group 735 records were recorded. The patient and procedural parameters are provided in Table 1.

**Table 1:** Procedure distribution.

**Characteristic**	**No capnography**	**With capnography**	**Difference**
	**(*N* = 666)**	**(*N* = 735)**	
	**Number (percent)**		***p*** **-value**
**Department**
Endoscopy	262 (39.3)	339 (46.1)	0.65
Interventional Cardiology	331 (49.7)	340 (46.3)	
Bronchoscopy	73 (11.0)	56 (7.6)	
**ASA classification**
ASA I	139 (20.9)	190 (25.9)	0.018
ASA II	337 (50.6)	368 (50.1)	
ASA III	183 (27.5)	176 (24.0)	
ASA IV	7 (1.1)	1 (0.1)	
**Sedative**
Midazolam	116 (17.4)	119 (16.2)	0.81
Fentanyl	33 (5.0)	27 (3.7)	
Midazolam + fentanyl	393 (59.0)	473 (64.4)	
Pethidine	1 (0.2)	1 (0.1)	
Propofol	3 (0.5)	1 (0.1)	
Diazepam	80 (12.0)	95 (12.9)	
Diazepam + fentanyl	40 (6.0)	19 (2.6)	
**Supplemental oxygen**
Yes	411 (61.7)	453 (61.6)	0.94
No	255 (38.3)	282 (38.4)	

*ASA, American Society of Anesthesiologists*.

*Absolute patient count in each category*.

There were differences between the baseline and capnography groups. The highest relative deviation between the groups was found in the department contributions. Due to a lower referral rate for bronchoscopy during the capnography data collection, their contribution was reduced and additional procedural data for endoscopy was collected. This led to a 7.8%-point increase in endoscopy-related procedures contributing to the capnography dataset compared to baseline. In both groups, the majority of patients were classed as ASA 2, with 50.6% in baseline vs. 50.1% in the capnography group. The fraction of ASA 1 patients increased from 20.9% in the baseline to 25.9% in the capnography group (Table 1). Given the difference in the distribution of ASA classifications the populations were found to be significantly different (*p* = 0.018) In terms of sedatives used, the predominant mode of sedation was a combination of midazolam and fentanyl (59.0% at baseline vs. 64.4% for capnography). The use of peri-operative supplemental oxygen was equivalent between both groups.

The aim of this SE was to quantify the impact of addition of capnography monitoring to standard practice at CUH on the incidence of sedation-related adverse events. The baseline group reported a total of 147 SIVA-defined adverse events (22.1 events per 100 procedures) compared with 93 adverse events (12.7 events per 100 procedures) in the capnography group. The reduction in these adverse events was 43.2% with use of capnography. This would be equivalent to one event prevented per 10.6 procedures. More than one event could be recorded for a single patient if that event was classed in a different category. Therefore, on a patient level, 135/666 (20.3%) of procedures had at least one adverse event in the baseline group and 87/735 (11.8%) of procedures had at least one adverse event in the post-capnography group.

Considering only the four adverse events as defined in the primary endpoint the SE target of a 20% reduction was surpassed, with a statistically significant 42.0% (95% CI: 23.0–60.7%, *p* <0.05) reduction in the incidence with use of capnography.

Examining the adverse event profiles, there were 132 low risk, 4 moderate risk, and 11 high-risk adverse events in the baseline group (Table 2). In the capnography group, this was 82 low risk, one moderate risk, and 10 high-risk adverse events. The 10 high-risk adverse events included one patient death which was, following an independent root-cause analysis, deemed unrelated to the usage of the capnography technology. There were 5 minor cardiac events recorded in the capnography group compared to 26 in the baseline group.

**Table 2:** Recorded events.

**Event**	**Low risk**	**Moderate risk**	**High risk**	**Total**
	**Number (% of procedures with ≥1 event)**			
**Capnography** ***n*** **= 735**
Total adverse events	82 (11.2%)	1 (0.1%)	10 (1.4%)	93 (12.7%)
Respiratory*	77 (10.5%)	–	7 (1.0%)	
Cardiac^†^	5 (0.7%)	–	2 (0.3%)	
Outcomes^‡^	–	1 (0.1%)	1 (0.1%)	
**Intervention** ^**#**^
Pre-defined	0	6	1	
Open text	44	0	0	
All	44 (6.0%)	6 (0.8%)	1 (0.1%)	
**Baseline** ***N*** **= 666**
Total adverse events	132 (19.8%)	4 (0.6%)	11 (1.7%)	147 (22.1%)
Respiratory*	106 (15.9%)	–	10 (1.5%)	
Cardiac^†^	26 (3.9%)	–	1 (0.2%)	
Outcomes^‡^	–	4 (0.6%)	0	
**Intervention** ^**#**^
Pre-defined	0	12	1	
Open text	56	0	2	
All	56 (8.4%)	12 (1.8%)	3 (0.5%)	

*Absolute count of adverse events by SIVA-assigned risk classification (11). Low, moderate, and high risk correspond with minimal, low, and sentinel risk as introduced by Mason et al. (11)*.

* *Pre-defined respiratory events: oxygen desaturation, severe oxygen desaturation, prolonged apnoea, airway obstruction*.

† *Pre-defined cardiac events: bradycardia, tachycardia, cardiovascular shock/collapse, cardiac arrest/absent pulse*.

‡ *Pre-defined outcomes: escalation of care, patient death*.

# *Pre-defined interventions: chest compressions, tracheal intubation, oral/nasal airway, bag valve mask/assisted ventilation, laryngeal mask airway, CPAP, reversal agents*.

To estimate the associated risk of having an adverse event with capnography monitoring, odds ratios were calculated (Table 3). Overall, a significant reduction in adverse events was found when capnography monitoring was implemented, the odds ratio being 0.50 (95% CI: 0.38–0.67). A significant reduction was also observed at a clinical service level for endoscopy and IC. Bronchoscopy saw a reduction in the overall adverse event risk, but the results did not reach statistical significance.

**Table 3:** Unadjusted odds ratio for respiratory compromise by ASA level and department after implementation of capnography.

	**ASA risk classification**			
	**Odds ratio (95% CI)**			
	**ASA I**	**ASA II**	**ASA III**	**All ASA**
Overall	**0.39 (0.21–0.72)**	0.68 (0.45–1.02)	**0.42 (0.24–0.73)**	**0.50 (0.38–0.67)**
**Department**			
Endoscopy	0.71 (0.24–2.13)	0.41 (0.13–1.24)	**0.24 (0.06–0.94)**	**0.32 (0.16–0.64)**
Interventional cardiology	0.46 (0.15–1.4)	**0.61 (0.37–1)**	0.88 (0.47–1.63)	**0.57 (0.40–0.82)**
Bronchoscopy	0.34 (0.06–1.79)	**3.52 (1.06–11.64)**	1.13 (0.26–5.01)	0.77 (0.36–1.63)

*Odds ratios indicated in **bold** font are considered significant at the 95% level of confidence (95% CI), p < 0.05*.

To reduce the effect of confounding variables on the prediction of adverse events, a multivariate linear regression analysis was performed (Table 4). Of the parameters assessed, clinical services for IC and bronchoscopy were associated with the highest increase in adverse-event risk. Capnography use was the only factor associated with a significant reduction in adverse-event risk.

**Table 4:** Multivariate linear regression model.

	**Estimate**	***p*** **-value**	**Adjusted odds ratio**
Department, interventional cardiology	2.33	*P* <0.001	10.30 (6.46, 16.60)
Department, bronchosopy	1.92	*P* <0.001	6.84 (4.13, 11.30)
Use of supplemental oxygen	1.19	*P* <0.001	3.29 (2.19, 4.93)
ASA III	0.87	*P* <0.001	2.39 (1.54, 3.77)
Use of capnography	−0.568	*P* <0.001	0.57 (0.42, 0.77)

*ASA, American Society of Anesthesiologists*.

*Only parameters identified by the multivariate linear regression analysis identified by p-value to be significant are shown. Parameters are sorted in order of increasing p-value. The estimate column contains the estimated model coefficient. Negative values indicate those that predict a decreased likelihood of an adverse event. Adjusted odds ratios for the odds of an adverse event for input parameters are shown along with 95% confidence intervals. Odds ratio values below 1 indicate association with decreased odds of an adverse event, values above 1 indicate an association with increased odds of an adverse event*.

Adjusted odds ratios (Table 4) were calculated, incorporating the population differences as determined by the multivariate regression model. The overall odds ratio for an adverse event with capnography usage was 0.57 (95% CI: 0.42–0.77) only slightly higher than in the unadjusted odds ratio of 0.50 and still statistically significant.

## Discussion

Overall, a positive safety benefit with capnography monitoring was observed. The primary clinical objective was surpassed, with an observed 42.0% reduction in total adverse events. The adjusted odds ratio calculation showed the use of capnography was a significant independent predictor of a reduction in adverse events after accounting for department, ASA classification, and use of supplementary oxygen. The physicians of the participating departments considered the addition of this monitoring system to provide additional confidence to their sedation approach, which is mostly physician-led in the UK NHS. While most observed events were classified as low risk events, the early detection and timely intervention is considered important for the prevention of progression to higher-risk adverse events. To confirm whether capnography monitoring could aid in reducing high-risk events, a study involving larger numbers of patients is required. Evidence from meta-analysis suggests, though, that use of capnography monitoring significantly reduces the incidence of patients requiring bag-mask ventilation (12).

Given previous literature (12), a reduction in adverse events was the expected result. However, the size of the impact that capnography made in a SE, or real-world setting, is larger than the Safe-Sedation Committee anticipated. They are, though, in line with a recent publication on endoscopy procedures from Belgium (14). There are still open questions on risk stratification and patient prioritization for capnography monitoring in our hospital. Results from our SE, specifically the adjusted odds ratio calculation, commend further investigation. Adverse events are more common in IC and bronchoscopy, but when stratified by department only in the IC and endoscopy departments did the impact of capnography reach significance (Table 4). The use of capnography in ASA III patients also requires further examination given data collected as part of our SE.

Not included in the data collected but reported by the participating physicians was a perceived increase in the ability to successfully complete more complex procedures. It is assumed that use of capnography monitoring increased the confidence of physicians to push through prolonged procedures without jeopardizing the patient's health. Whether this is true needs to be confirmed in future prospective clinical trials. Successful completion of procedures was not included in collected data and we recommend that it to be added to any future SE of capnography monitoring.

A convenience sample of patients was used in this SE because nursing staff trained and qualified in the use of capnography were required to be present for a procedure to be included in the analysis. Training and familiarity with capnography may, therefore, be an important aspect in the benefit identified during this SE. The learning curve is a recognized part of the introduction of any type of medical device. Most adverse events require one or more interventions, consuming both time and resources. The impact on healthcare costs may be considerable given the difference in adverse event incidence between the two groups. The most common respiratory event, mild oxygen desaturation, has been associated with a median cost increase of GBP 20 (11–28) (7). Cost collection during this SE was out of scope, but could be of interest to other providers.

This SE is one of the first to explore applications of capnography beyond endoscopy. As far as we are aware, this evaluation provides the first results on capnography usage in bronchoscopy and IC. For both bronchoscopy and IC departments, this SE showed promising results with IC reporting reduced adverse-event risks for all ASA classifications and bronchoscopy.

## Limitations

This SE may not be considered as a full clinical study of the efficacy of capnography monitoring. The patients were not randomized, and the physicians could not be blinded for the method. The non-controlled design and the distribution over three participating services made it additionally difficult to reach full adequacy in the distribution between the baseline and capnography group. In addition, the serial design, where the baseline data were collected before the capnography data may have introduced confounding effects to the data due to differences in practice or experience that could not be controlled in the present analyses. In the present evaluation, some patients seen in the departments involved would not have been captured in our collected data due to practical difficulties. During peaks of activity and personnel shortage, it was not feasible to collect data on all procedures conducted. Therefore, the patient sample is neither strictly prospective nor cumulative but an opportunistic representation. However, a sufficiently powered sample size and reduction in adverse events reflects an improvement in everyday practice and patient safety. Clinical practice varies by hospital and the utility of capnography depends on trained staff, as such results might not be fully reproducible in other settings. Still similar benefits of capnography to those reported here have been found by other groups (2, 9, 14).

## Conclusion

Our SE showed that the adoption of capnography for selected procedures may lead to an increase of patient safety during procedural sedation. The incidence of adverse events was significantly reduced by 43.2% (*p* < 0.05). The resulting adjusted odds ratio for the target adverse events was 0.57 (95% CI: 0.42–0.77). The authors would support adding capnography to the minimal monitoring standards for the subset of procedures as analyzed in this publication.

## Data Availability Statement

The dataset for this study can be made available in an anonymised fashion upon reasonable request. Requests to access the datasets should be directed to RT, enquiries@coreva-scientific.com.

## Ethics Statement

Ethical approval was not provided for this study on human participants because the hospital supported the integration of capnography monitoring as an established, non-experimental element of procedural sedation. There was no prospective patient assignment or randomization, and so the addition was classified as a service evaluation and not a clinical study. On assessment by the participating departments, Ethics Committee approval was deemed not required but the evaluation project is registered with the hospitals Clinical Audit Department and results reported in line with their requirements. Written informed consent for participation was not required for this study in accordance with the national legislation and the institutional requirements.

## Author Contributions

GC, PP, JH, TS, DM-R, and AP contributed to the research concept and conduct, and to reviewing and editing the manuscript. RT contributed to data analysis, reporting and drafting the manuscript. RS contributed to the conception, data analysis, reporting and drafting the manuscript. CL contributed to the conception, coordination, and reviewing the manuscript. All authors acknowledge full responsibility for the analyses and interpretation of the report and have read and approved the final manuscript.

## Conflict of Interest

RT is an employee of Coreva Scientific, and reports receiving consulting fees from Medtronic during the conduct of the study. RS is the owner of Coreva Scientific, and reports receiving consulting fees from Medtronic during the conduct of the study; RS also reports receiving consulting fees from Medtronic, Cardinal Health, and Medasense, outside the submitted work. CL is a full time employee of Medtronic UK. AP reports non-financial support from Medtronic, in form of provisioning of monitors and training during the conduct of the study. The remaining authors declare that the research was conducted in the absence of any commercial or financial relationships that could be construed as a potential conflict of interest. The authors declare that this study received funding from Medtronic. The funder had the following involvement in the study: provision capnography monitors and training on the use of capnography monitoring. CL, an author on this study and an employee of the funder, was involved in design of the study and interpretation of data. The funder was neither involved in the data collection, analysis, nor decision to submit it for publication. Because an employee of the funder was an author, the funder did review the manuscript prior to submission to ensure legal compliance - no changes were requested by the funder.

## Publisher's Note

All claims expressed in this article are solely those of the authors and do not necessarily represent those of their affiliated organizations, or those of the publisher, the editors and the reviewers. Any product that may be evaluated in this article, or claim that may be made by its manufacturer, is not guaranteed or endorsed by the publisher.
