# Corrigendum: Service evaluation of the impact of capnography on the safety of procedural sedation

**DOI:** 10.3389/fmed.2022.999862

**Published:** 2022-08-05

**Authors:** Gareth Corbett, Peter Pugh, Jurgen Herre, Teik Choon See, David de Monteverde-Robb, Rafael Torrejon Torres, Rhodri Saunders, Catherine Leonard, Amit Prakash

**Affiliations:** ^1^Department of Gastroenterology, Cambridge University Hospital National Health Service Trust, Cambridge, United Kingdom; ^2^Department of Cardiology, Cambridge University Hospital National Health Service Trust, Cambridge, United Kingdom; ^3^Department of Respiratory Medicine, Cambridge University Hospital National Health Service Trust, Cambridge, United Kingdom; ^4^Department of Radiology, Cambridge University Hospital National Health Service Trust, Cambridge, United Kingdom; ^5^Department of Pharmacy, Cambridge University Hospital National Health Service Trust, Cambridge, United Kingdom; ^6^Health Economics, Coreva Scientific, Koenigswinter, Germany; ^7^Health Economics, Medtronic UK Ltd., Watford, United Kingdom; ^8^Department of Anaesthesia, Cambridge University Hospital National Health Service Trust, Cambridge, United Kingdom

**Keywords:** safe sedation practice, monitoring, respiratory compromise, endoscopy, bronchoscopy, patient safety

In the published article, there was an error.

A correction has been made to the **Conclusion**. This sentence previously stated:

“The incidence of adverse events was significantly reduced by 53.2% (*p* <0.05).”

The corrected sentence appears below:

“The incidence of adverse events was significantly reduced by 43.2% (*p* <0.05).”

The authors apologize for this error and state that this does not change the scientific conclusions of the article in any way. The original article has been updated.

## Publisher's note

All claims expressed in this article are solely those of the authors and do not necessarily represent those of their affiliated organizations, or those of the publisher, the editors and the reviewers. Any product that may be evaluated in this article, or claim that may be made by its manufacturer, is not guaranteed or endorsed by the publisher.

