# Supplementary material for: Service Evaluation of the Impact of Capnography on the Safety of Procedural Sedation
Source: Front Med (Lausanne). 2022 May 6;9:867536. doi: 10.3389/fmed.2022.867536 (PMC9122489; doi:10.3389/fmed.2022.867536)
Supplement: Supplementary file 1 [file Data_Sheet_1.docx]

**Supplementary material**

**Table S1 Interventional cardiology procedure codes**

| Interventional cardiology |
| --- |
| [K611] Implantation of cardiac pacemaker system NEC |
| [K615] Implantation of single chamber cardiac pacemaker system |
| [K616] Implantation of dual chamber cardiac pacemaker system |
| [K634] Coronary arteriography using two catheters |
| [K633] Angiocardiography of left side of heart NEC |
| [K636] Coronary arteriography NEC |
| [K631] Angiocardiography of combination of right and left side of heart |
| [K632] Angiocardiography of right side of heart NEC |
| [K771] Percutaneous transluminal pericardiocentesis |
| [K651] Catheterisation of combination of right and left side of heart NEC |
| [K653] Catheterisation of left side of heart NEC |
| [K659] Unspecified catheterisation of heart |
| [U202] Transoesophageal echocardiography |
| [X501] Direct current cardioversion |
| [K611] Implantation of cardiac pacemaker system NEC |

**Table S2 Endoscopy procedure codes**

| Endoscopy |
| --- |
| [J391] Endoscopic sphincterotomy of accessory ampulla of vater |
| [J398] Other specified other therapeutic endoscopic operations on ampulla of vater |
| [J399] Unspecified other therapeutic endoscopic operations on ampulla of vater |
| [J171] Endoscopic ultrasound examination of liver and biopsy of lesion of liver |
| [J178] Other specified endoscopic ultrasound examination of liver |
| [J179] Unspecified endoscopic ultrasound examination of liver |
| [J531] Endoscopic ultrasound examination of bile duct and biopsy of lesion of bile duct |
| [J538] Other specified endoscopic ultrasound examination of bile duct |
| [J539] Unspecified endoscopic ultrasound examination of bile duct |
| [J741] Endoscopic ultrasound examination of pancreas and biopsy of lesion of pancreas |
| [J748] Other specified endoscopic ultrasound examination of pancreas |
| [J749] Unspecified endoscopic ultrasound examination of pancreas |

**Table S3 Respiratory medicine procedure codes**

| Bronchoscopy |
| --- |
| Bronchoscopy Diagnostic [E51] |
| Bronchoscopy Therapeutic [E50] |
| Bronchoscopy Therapeutic for removal of foreign object [E505] |

**Table S4 Interventional radiology procedure codes**

| Interventional radiology |
| --- |
| [J101] Percutaneous transluminal embolisation of hepatic artery |
| [J102] Percutaneous transluminal embolisation of portal vein |
| [J121] Percutaneous drainage of liver |
| [J131] Percutaneous transvascular biopsy of lesion of liver |
| [J212] Drainage of gall bladder |
| [J471] Percutaneous insertion of tubal prosthesis into both hepatic ducts |
| [J472] Percutaneous insertion of tubal prosthesis into right hepatic duct NEC |
| [J474] Percutaneous insertion of tubal prosthesis into hepatic duct NEC |
| [J475] Percutaneous insertion of tubal prosthesis into common bile duct |
| [J479] Unspecified therapeutic percutaneous insertion of prosthesis into bile duct |
| [J485] Percutaneous transhepatic biliary drainage multiple |
| [L433] Percutaneous transluminal embolisation of renal artery |
| [L435] Percutaneous transluminal insertion of stent into renal artery |
| [L474] Percutaneous transluminal insertion of stent into visceral branch of abdominal aorta NEC |
| [L713] Percutaneous transluminal embolisation of artery |
| [L943] Percutaneous transluminal insertion of subcutaneous port |
| [L945] Percutaneous transluminal insertion of stent into vein NEC |
| [L947] Percutaneous transluminal balloon angioplasty of vein NEC |

T**able S5 Vascular access procedure codes**

| Vascular access |
| --- |
| [L912] Insertion of central venous catheter NEC |
| [L915] Insertion of tunnelled venous catheter |
| [M335] Percutaneous insertion of ureteric stent into ureter NEC |
